# Trends in Early Childhood Obesity in a Large Urban School District in the Southwestern United States, 2007–2014

**DOI:** 10.5888/pcd13.150594

**Published:** 2016-06-02

**Authors:** Andrea Cantarero, Orrin Myers, Thomas Scharmen, Peter Kinyua, Elizabeth Yakes Jimenez

**Affiliations:** Author Affiliations: Orrin Myers, Elizabeth Yakes Jimenez, University of New Mexico, Albuquerque, New Mexico; Thomas Scharmen, New Mexico Department of Health, Santa Fe, New Mexico; Peter Kinyua, Albuquerque Public Schools, Albuquerque, New Mexico.

## Abstract

**Introduction:**

Although recent studies indicate that rates of childhood obesity and severe obesity may be declining, few studies have reported prevalence trends in early childhood or differences in trends across sociodemographic groups. The primary aim of this study was to report trends in prevalence of early childhood obesity and severe obesity 2007 through 2014 in a diverse, metropolitan school district in the southwestern United States and determine whether these trends vary by race/ethnicity, socioeconomic status, and disability status.

**Methods:**

We analyzed height, weight and demographic data from 43,113 kindergarteners enrolled in a large, urban school district in the southwestern United States for 7 school years. Adjusted odds of obesity and severe obesity were calculated to assess changes in prevalence for non-Hispanic white, Hispanic, and American Indian students; free or reduced-price lunch participants and nonparticipants; and students with and without disabilities. To test for differences in obesity trends, interaction terms were added to the logistic regressions between school year and sex, race/ethnicity, free or reduced-price lunch participation, and disability status.

**Results:**

The adjusted prevalence of both obesity (from 13.1% in 2007–2008 to 12.0% in 2013–20014) and severe obesity (from 2.4% in 2007–2008 to 1.2% in 2013–2014) declined overall. We found no significant interactions between the adjusted prevalence of obesity over time and any of the sociodemographic subgroups. Obesity prevalence declined more among American Indian students than among Hispanic or non-Hispanic white students.

**Conclusion:**

In this district, from 2007 through 2014, severe obesity decreased and obesity did not increase, overall and across all sociodemographic subpopulations for kindergarten students.

## Introduction

Childhood obesity is a major public health concern in the United States and is the focus of many policy and programmatic interventions. Timely and demographically representative monitoring of obesity prevalence is essential to effectively address childhood obesity locally and nationally (1,2). Few obesity surveillance systems exist for children aged 5 to 11 years, who are too young to participate in the Youth Risk Behavior Surveillance System and too old to be eligible for the Special Supplemental Nutrition Program for Women, Infants, and Children (WIC). To track and address childhood obesity at state and local levels, school-based surveillance programs have become more common (2). However, few communities have published their data on obesity prevalence among elementary school children (1).

National evidence indicates that the childhood obesity epidemic has finally plateaued, with significant declines in obesity prevalence in recent years for children aged 2 to 5 years (3) and low-income children aged 2 to 4 years (4). However, neither of these studies had sufficient sample size to investigate trends in the American Indian population, nor did they make direct comparisons by sociodemographic characteristics such as socioeconomic status (SES) or disability status. Emerging evidence indicates that children with some disabilities are also at increased risk of obesity (5–8). Disparities in childhood obesity by race/ethnicity and SES are well-documented (3,9–12) and widening (13–16). Severe obesity has emerged as a distinct class of pediatric obesity that should be a priority for public health surveillance (17,18), and it differs significantly by both race/ethnicity (19–22) and SES (20).

The primary aim of this study was to report trends in prevalence of childhood obesity and severe obesity 2007 through 2014 from an ethnically diverse, metropolitan school district in the southwestern United States. The secondary aim was to determine whether these trends vary by race/ethnicity, SES, and disability status.

## Methods

This retrospective cohort study was conducted using secondary, de-identified data collected from 2007 through 2014 in a metropolitan school district in the southwestern United States that serves more than 90,000 students. The school district research review board and the University of New Mexico Human Research Protections Office reviewed and approved the study protocol before the de-identified data set was shared.

School nurses collected data on the heights and weights of all kindergarten students annually from 2007 through 2014 as part of a district-wide obesity surveillance project. Height was measured to the nearest 1/8 inch using wall-mounted stadiometers, and weight was measured to the nearest 1/4 pound using calibrated beam balance scales. Measurement dates were recorded. All school nurses in the district received training on measuring student heights and weights as well as on balance beam calibration during a series of workshops from 2007 through 2009. A district resource nurse was responsible for training all new school nurses on the measurement protocol and was responsible for continuous quality assurance of measurements collected by nurses through 2010. Since then, annual height and weight measurement training has been offered to the district’s school nurses by the state department of health as part of a broader statewide obesity surveillance project.

Student identification numbers were used by school district staff to link anthropometric data to other student information: sex, birth date, race/ethnicity, school enrollment, free or reduced-price lunch (FRPL) status (as a proxy for SES), and disability status. Disability included any of the following special education designations: intellectual disability, hearing impairment, speech or language impairment, visual impairment, emotional disturbance, orthopedic impairment, learning disability, deaf-blindness, autism, traumatic brain injury, developmental delay, other health impairment, and multiple disabilities. Data on student race/ethnicity were collected on the basis of parent report during enrollment. Students enrolled in multiple schools in a single year were assigned to a single school of enrollment on the basis of greatest proportion of attendance. Student identification numbers were removed and a study identification number was randomly assigned to each student before the district released the data to the research team.

Birth dates and examination dates were used to calculate age, which was combined with data for height, weight, and sex to compute body mass index (BMI) percentiles and to identify biologically implausible values (BIVs) using Statistical Analysis Software (SAS) codes provided by the Centers for Disease Control and Prevention (CDC) (23). Children were assigned to a weight status category of underweight (BMI <5th percentile), normal weight (BMI ≥5th percentile and <85th percentile), overweight (BMI ≥85th percentile and <95th percentile) or obese (≥95th percentile) based on CDC growth chart criteria (18). Severe obesity was defined as a BMI that is either greater than 35 or 120% of the 95th percentile, whichever is lower, based on guidance from the American Heart Association (17).

**Figure 1:**
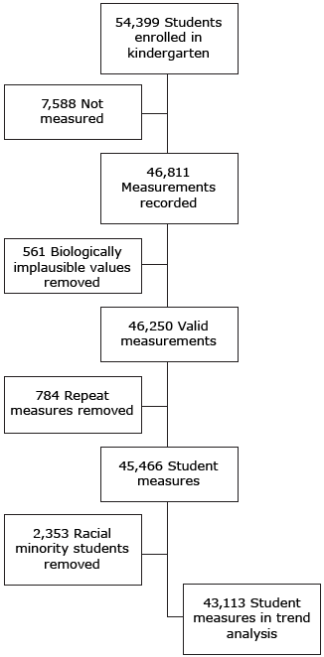
Measurements of kindergarten students and final analytic sample, Urban School District in the Southwestern United States, 2007–2014.

Only the first biologically plausible measurement for each student was used in the analytic data set. On the basis of these criteria, 43,113 measures were included in the analysis (Figure 1); 561 BIVs and 784 repeated measurements (primarily from kindergarten students who were held back a grade) were excluded from analysis. For the purpose of analyzing obesity prevalence trends over time, each student was included in one of the following racial/ethnic groups: non-Hispanic white, American Indian (any ethnicity), or Hispanic (any non–American Indian race). Because of small sample sizes, data for the following non-Hispanic races were excluded from analysis: black or African American, Asian American, and Native Hawaiian or Pacific Islander.

Data analysis was completed using SAS Version 9.4 (SAS Institute Inc). Generalized estimating equation models were used to calculate unadjusted and adjusted odds of obesity and severe obesity to appropriately control for the clustering of children from 94 different public elementary schools. The prevalence and unadjusted bivariate odds of obesity and severe obesity were calculated for age (<6 and ≥6), sex (male/female), race/ethnicity (non-Hispanic white, Hispanic [any non–American Indian race] American Indian [any ethnicity]), FRPL participation (no/yes), disability (no/yes), school year (2007–2014, at one-year intervals), and examination date (January through December, at 2-month intervals). Adjusted odds ratios were calculated using multivariable models adjusting for child age (as a continuous variable), sex, race/ethnicity, FRPL participation, disability, and exam date. School years were analyzed as categorical, for comparisons against the baseline school year, and as continuous variables, to test for a linear trend over the entire study period. To test for differences in obesity trends by sociodemographic groups, interaction terms were added to the logistic regressions between school year and sex, race/ethnicity, FRPL participation, and disability status. Wald χ^2^ tests were used to calculate 95% confidence intervals. Significance was determined at *P* < .05.

## Results

Approximately 80% of all kindergarten students enrolled in the district from 2007 through 2014 met our inclusion criteria. Overall, the demographics of the measured sample were similar to the enrolled population in the school district (Table 1). Most of the included students were at a normal weight, 5 years old, Hispanic, enrolled in FRPL, and measured during the fall. The overall prevalence of obesity was 12.1% throughout the study period.

**Table 1:** Demographic Characteristics^a^ of Kindergarteners in the Study Sample (n = 43,113) and the Metropolitan Southwestern United States School District (N = 54,399), 2007–2014

Characteristic	Study Sample	School District
	No. (%)	
**Weight class^b^**		
Underweight	1,557 (3.6)	NA
Normal weight	30,128 (69.9)	NA
Overweight	6,218 (14.4)	NA
Obese	4,157 (12.1)	NA
Severely obese	1,053 (2.4)	NA
**Age, y**		
≤4	257 (0.6)	275 (0.6)
5	32,539 (75.5)	34,739 (74.2)
≥6	10,317 (23.9)	11,783 (25.2)
**Sex**		
Male	21,726 (50.4)	28,505 (50.8)
Female	21,387 (49.6)	27,281 (49.2)
**Race/ethnicity**		
Hispanic (any non–American Indian race)	29,980 (69.5)	35,929 (69.7)
Non-Hispanic white	10,474 (24.3)	12,272 (23.8)
American Indian (any ethnicity)	2,659 (6.2)	3,360 (6.5)
**Free or reduced-price lunch participant**		
Yes	23,040 (67.0)	36,001 (66.7)
No	14,165 (33.0)	17,998 (33.3)
**Disability^c^**		
Yes	2,595 (6.0)	3,824 (6.6)
No	40,518 (94.0)	51,963 (93.4)
**School year**		
2007–2008	5,507 (12.8)	8,000 (14.3)
2008–2009	5,883 (13.7)	7,853 (14.1)
2009–2010	6,190 (14.4)	7,801 (14.0)
2010–2011	6,534 (15.2)	7,923 (14.2)
2011–2012	6,254 (14.5)	7,931 (14.2)
2012–2013	6,554 (15.2)	8,280 (14.8)
2013–2014	6,191 (14.4)	7,999 (14.3)
**Examination date**		
January–February	5,892 (13.7)	NA
March–April	2,907 (6.7)	NA
May–June	691 (1.6)	NA
July–August	2,855 (6.6)	NA
September–October	15,933 (37.0)	NA
November–December	14,835 (34.4)	NA

Abbreviations: BMI, body mass index; CDC, Centers for Disease Control and Prevention; NA, not applicable.

^a^ Values may not sum to total due to missing data.

^b^ Weight class categories defined as underweight (BMI <5th percentile), normal weight (BMI ≥5th percentile and <85th percentile), overweight (BMI ≥85th percentile and <95th percentile), or obese (≥95th percentile) based on percentiles from CDC sex-specific BMI-for-age growth charts. Severely obese was defined as a BMI above 35 kg/m^2^ or 120% of the 95th percentile, whichever was lower, based on CDC sex-specific BMI-for-age growth charts. On the basis of these criteria, severely obese is a subset of obese.

^c^ Disability included any of the following special education designations: intellectual disability, hearing impairment, speech or language impairment, visual impairment, emotional disturbance, orthopedic impairment, learning disability, deaf-blindness, autism, traumatic brain injury, developmental delay, other health impairment, and multiple disabilities.

On the basis of bivariate analyses (Table 2), male sex, Hispanic ethnicity, American Indian race, and FRPL enrollment were all positively associated with obesity. Age, Hispanic ethnicity, American Indian race, and FRPL enrollment were significantly associated with severe obesity. Compared with non-Hispanic white students, American Indian students had the highest odds of being both obese and severely obese, followed by Hispanic students. No clear pattern was evident for a relationship between examination date and obesity or severe obesity, and no significant trends were found in the prevalence of unadjusted obesity (*P* = .68) or unadjusted severe obesity (*P* = .18) during this period.

**Table 2:** Bivariate Relationships Between Student Characteristics and Odds of Obesity and Severe Obesity in a Kindergarten Sample (n = 43,113) From a Metropolitan School District in the Southwestern United States, 2007–2014

Characteristic	Obesity^a^			Severe Obesity^a^		
	Prevalence, n (%)	OR (95% CI)	*P* Value^b^	Prevalence, n (%)	OR (95% CI)	*P* Value^b^
**Age, y**						
<6	3,910 (11.9)	1 [Reference]	.10	730 (2.2)	1 [Reference]	<.001
≥6	1,300 (12.6)	1.06 (0.99–1.14)		323 (3.1)	1.42 (1.25–1.63)	
**Sex**						
Female	2,442 (11.4)	1 [Reference]	<.001	520 (2.4)	1 [Reference]	.82
Male	2,768 (12.7)	1.14 (1.07–1.21)		533 (2.5)	1.01 (0.90–1.14)	
**Race/ethnicity**						
Non-Hispanic white	702 (6.7)	1 [Reference]	<.001	115 (1.1)	1 [Reference]	<.001
Hispanic (any non–American Indian race)	3,986 (13.3)	1.79 (1.66–1.94)	<.001	820 (2.7)	2.19 (1.82–2.64)	<.001
American Indian (any ethnicity)	522 (19.6)	2.96 (2.58–3.39)	<.001	118 (4.4)	3.65 (2.68–4.97)	<.001
**Free or reduced-price lunch participant**						
No	1,227 (8.7)	1 [Reference]	<.001	211 (1.5)	1 [Reference]	<.001
Yes	3,961 (13.8)	1.40 (1.25–1.55)		837 (2.9)	1.73 (1.43–2.08)	
**Disability^c^**						
No	4,867 (12.0)	1 [Reference]	.07	984 (2.4)	1 [Reference]	.46
Yes	343 (13.2)	1.12 (1.00–1.27)		69 (2.7)	1.10 (0.86–1.41)	
**School year**						
2007–2008	655 (11.9)	1 [Reference]	.34	136 (2.5)	1 [Reference]	.02
2008–2009	691 (11.8)	0.96 (0.86–1.07)	.47	130 (2.2)	0.88 (0.71–1.10)	.26
2009–2010	822 (13.3)	1.09 (0.97–1.24)	.15	174 (2.8)	1.12 (0.90–1.38)	.31
2010–2011	789 (12.1)	0.99 (0.88–1.10)	.81	171 (2.6)	1.04 (0.81–1.33)	.75
2011–2012	719 (11.5)	0.95 (0.83–1.08)	.42	156 (2.5)	1.00 (0.80–1.26)	1.00
2012–2013	793 (12.1)	0.99 (0.85–1.16)	.94	173 (2.6)	1.05 (0.81–1.37)	.71
2013–2014	741 (12.0)	0.99 (0.87–1.13)	.88	113 (1.8)	0.73 (0.56–0.93)	.01
**Examination date**						
January–February	723 (12.3)	1 [Reference]	.14	155 (2.6)	1 [Reference]	.73
March–April	394 (13.6)	1.16 (1.03–1.31)	.02	86 (3.0)	1.15 (0.86–1.54)	.34
May–June	79 (11.4)	1.10 (0.87–1.39)	.44	20 (2.9)	1.25 (0.80–1.95)	.32
July–August	361 (12.6)	0.98 (0.83–1.17)	.83	69 (2.4)	0.86 (0.60–1.22)	.39
September–October	1,822 (11.4)	1.04 (0.94–1.15)	.47	366 (2.3)	0.94 (0.75–1.19)	.62
November–December	1,831 (12.3)	1.11 (1.00–1.24)	.05	357 (2.4)	0.96 (0.79–1.16)	.66

Abbreviations: BMI, body mass index; CDC, Centers for Disease Control and Prevention; CI, confidence interval; OR, odds ratio.

^a^ Obese defined as BMI at or above the 95th percentile on the CDC sex-specific BMI-for-age growth charts. Severely obese defined as BMI above 35 kg/m^2^ or 120% of the 95th percentile, whichever was lower, based on CDC sex-specific BMI-for-age growth charts.

^b^ Overall *P* values (listed in reference group for characteristics with more than 2 categories) calculated using Wald χ^2^ test for type 3 analysis of effects. Within-category *P* values and 95% CIs calculated using Wald χ^2^ test. ORs were adjusted for clustering by school.

^c^ Disability included any of the following special education designations: intellectual disability, hearing impairment, speech or language impairment, visual impairment, emotional disturbance, orthopedic impairment, learning disability, deaf-blindness, autism, traumatic brain injury, developmental delay, other health impairment, and multiple disabilities.

In the final adjusted models (Table 3), Hispanic ethnicity, American Indian race, and FRPL enrollment remained significantly and positively associated with both obesity and severe obesity (*P* <.001). Older age was a significant risk factor for severe obesity (*P* < .001) but not obesity (*P* = .12), and male sex was a significant risk factor for obesity (*P* < .001) but not severe obesity (*P* = .88). Students measured during November and December had a slightly increased odds of obesity compared with students measured in January and February (*P* = .04).

**Table 3:** Multivariate Relationships Between Student Characteristics and Odds of Obesity and Severe Obesity in a Kindergarten Sample (n = 43,113) From a Metropolitan School District in the Southwestern United States, 2007–2014

Characteristic	Obesity^a^		Severe Obesity^a^	
	AOR (95% CI)	*P* Value^b^	AOR (95% CI)	*P* Value^b^
**Age, y**	1.08 (0.98–1.19)	.12	1.81 (1.48–2.21)	<.001
**Sex**				
Female	1 [Reference]	<.001	1 [Reference]	.88
Male	1.14 (1.07–1.21)		1.01 (0.89–1.14)	
**Race/ethnicity**				
Non-Hispanic white	1 [Reference]	<.001	1 [Reference]	<.001
Hispanic (any non–American Indian race)	1.73 (1.60–1.88)	<.001	2.05 (1.67–2.51)	<.001
American Indian (any ethnicity)	2.83 (2.47–3.23)	<.001	3.32 (2.38–4.62)	<.001
**Free or reduced-price lunch participant**				
No	1 [Reference]	<.001	1 [Reference]	<.001
Yes	1.26 (1.14–1.40)		1.51 (1.25–1.83)	
**Disability^c^**				
No	1 [Reference]	.24	1 [Reference]	.84
Yes	1.08 (0.95–1.22)		1.03 (0.79–1.33)	
**School year**				
2007–2008	1 [Reference]	.21	1 [Reference]	.006
2008–2009	.97 (0.90–1.05)	.52	0.91 (0.78–1.06)	.20
2009–2010	1.09 (1.01–1.18)	.04	1.13 (0.98–1.31)	.10
2010–2011	0.99 (0.92–1.07)	.79	1.07 (0.91–1.26)	.40
2011–2012	0.94 (0.87–1.02)	.13	1.02 (0.89–1.17)	.82
2012–2013	0.98 (0.89–1.08)	.67	1.07 (0.89–1.27)	.48
2013–2014	0.97 (0.89–1.05)	.40	0.73 (0.61–0.88)	<.001
**Examination date**				
January–February	1 [Reference]	.33	1 [Reference]	.72
March–April	1.12 (0.99–1.26)	.07	1.00 (0.75–1.32)	.98
May–June	1.02 (0.80–1.30)	.87	1.04 (0.66–1.64)	.87
July–August	1.01 (0.84–1.21)	.93	1.15 (0.83–1.61)	.41
September–October	1.07 (0.96–1.20)	.24	1.19 (0.94–1.50)	.15
November–December	1.12 (1.01–1.25)	.04	1.10 (0.91–1.33)	.32

Abbreviations: AOR, adjusted odds ratio; BMI, body mass index; CDC, Centers for Disease Control and Prevention; CI, confidence interval, FRPL, free or reduced price lunch.

^a^ Obese defined as a BMI at or above the 95th percentile based on CDC sex-specific BMI-for-age growth charts. Severely obese defined as a BMI above 35 kg/m^2^ or 120% of the 95th percentile, whichever was lower, based on CDC sex-specific BMI-for-age growth charts.

^b^ Overall *P* values (listed in reference group for characteristics with more than 2 categories) calculated using Wald χ^2^ test for type 3 analysis of effects. Within-category *P* values and 95% CIs calculated using Wald χ^2^ test. ORs were simultaneously adjusted for clustering by school and all other variables shown in the table.

^c^ Disability included any of the following special education designations: intellectual disability, hearing impairment, speech or language impairment, visual impairment, emotional disturbance, orthopedic impairment, learning disability, deaf-blindness, autism, traumatic brain injury, developmental delay, other health impairment, and multiple disabilities.

The adjusted prevalence of both obesity (from 13.1% in 2007–2008 to 12.0% in 2013–2014) and severe obesity (from 2.4% in 2007–2008 to 1.2% in 2013–2014) declined overall during the study period. However, only the increase in obesity in 2009–2010 (*P* = .04) and decrease in severe obesity in 2013–2014 (*P* < .001) were significant compared with the baseline year of 2007–2008. The linear trend for this period indicated a significant decrease in severe obesity (*P* = .03), but not for obesity (*P* = .14).

**Figure 2:**
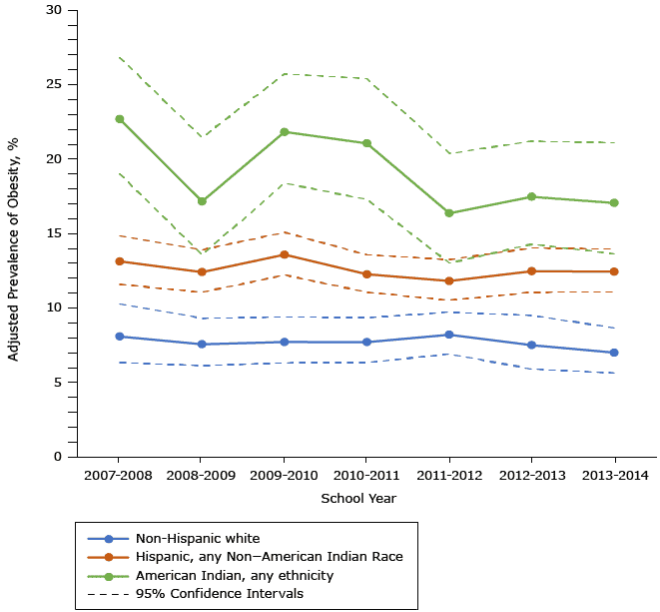
Adjusted annual prevalence of obesity, by race/ethnicity and school year, in a sample of kindergarten students in an Urban School District in the Southwestern United States, 2007–2014. Data adjusted for sex, free- or reduced-price lunch status, disability status, measurement date, and clustering by school. **Table:** 

No significant interactions were found between the adjusted prevalence of obesity over time and any of the sociodemographic subgroups. However, obesity prevalence declined in American Indian students from a high of 22.7% in 2007–2008 to 17.0% in 2013–2014, a 25.1% decrease. In contrast, the prevalence of obesity declined in non-Hispanic white students by 13.5% between 2007–2008 and 2013–2014 (8.1% to 7.0%), while the prevalence declined among Hispanic students by only 5.3% during the same period (13.1% to 12.4%) (Figure 2).

School Year	Race/Ethnicity, % (95% Confidence Interval)		
	Non-Hispanic White (n = 10,474)	Hispanic, Any Non–American Indian Race (n = 29,980)	American Indian, Any Ethnicity (n = 2,659)
2007–2008	8.1 (6.3–10.3)	13.1 (11.6–14.8)	22.7 (19.0–26.8)
2008–2009	7.5 (6.1–9.3)	12.4 (11.0–13.9)	17.1 (13.6–21.4)
2009–2010	7.7 (6.3–9.4)	13.6 (12.2–15.1)	21.8 (18.4–25.7)
2010–2011	7.7 (6.3–9.3)	12.2 (11.0–13.6)	21.1 (17.3–25.4)
2011–2012	8.2 (6.9–9.7)	11.8 (10.5–13.2)	16.4 (13.0–20.4)
2012–2013	7.5 (5.9–9.5)	12.5 (11.0–14.0)	17.5 (14.3–21.2)
2013–2014	7.0 (5.6–8.6)	12.4 (11.1–13.9)	17.0 (13.6–21.1)

## Discussion

Obesity did not increase and severe obesity decreased from 2007 through 2014 among kindergarteners in this racially/ethnically diverse metropolitan school district in the southwestern United States. This trend was similar for both sexes, all racial/ethnic groups, FRPL participants and nonparticipants, and students with and without disabilities. American Indian and Hispanic students had a higher prevalence of obesity and severe obesity than did non-Hispanic white students during the study period, despite the greatest declines in obesity prevalence being evident among American Indian students.

These findings are consistent with those of other national and regional reports, indicating that the childhood obesity epidemic has finally plateaued (3,4,20–22,24–27) and even decreased in certain populations (3,4,20,22,24,25,27). Four of these studies reported on obesity in kindergarteners (20,24–26), 2 reported on American Indian students (24,26), and only one reported on severe obesity in kindergarteners (20). The overall prevalence of obesity in our sample of kindergarteners, 12.1%, was similar to the finding of a surveillance report of kindergarteners in New Mexico, 11.6% in 2014 (24), which was somewhat lower than the prevalence for kindergarteners in public schools in Arkansas (15.9% in 2013–2014) (26) and much lower than the prevalence for kindergarteners in public schools in New York City (18.2% in 2010–2011) (20,25). Severe obesity was also more common in New York City (3.7% in 2010–2011) (20) than in our sample (2.4% in 2007–2014). Regional variation in childhood obesity in the United States is well documented and may be the result of cultural differences in nutrition and physical activity habits or differences in the home, early child education (28), school and community policies, and environments related to nutrition and physical activity (4).

To our knowledge, only one school-based surveillance project tested for trends in obesity in kindergarteners (20,25). New York City public schools reported significant declines in both obesity and severe obesity between 2006–2007 and 2010–2011 in kindergarteners (20). Day et al reported a 14.0% decrease in obesity and 9.9% decrease in severe obesity, compared with 8.4% and 50.0% in our sample of kindergarteners, respectively. The overall decline in obesity in our sample was not significant, which may have been the result of reduced power in our sample, given the small number of kindergarteners (approximately 43,000) compared with the New York City sample (approximately 669,000). Among the broader student population (kindergarten through 8th grade), significant declines in obesity and severe obesity were reported among both sexes and all racial/ethnic groups, as well as among FRPL participants and non-participants (20). However, Day et al noted that declines in obesity and severe obesity were greatest among the lowest risk populations (ie, non-Hispanic white and non–FRPL-participating students). In contrast, our findings show that American Indian students experienced greater relative and absolute declines in obesity (24.9% and 5.7%, respectively) than either Hispanic (5.3% and 0.7%) or non-Hispanic white students (13.5% and 1.1%), although these differences were not significant. Additionally, FRPL participation was not associated with a difference in obesity trends in our population. There are several reasons why our findings may differ from those in New York City, including regional differences in the experience of racial/ethnic minorities in the Southwest and differences in effectiveness of and access to community-wide and district-wide obesity prevention and treatment efforts.

Although it is impossible to determine the cause of these observed trends from the data available, several recent public health changes may be affecting early childhood obesity incidence and prevalence. At the federal level, WIC implemented multiple changes designed to make food packages healthier for participating women and children starting in late 2007, and the Healthy, Hunger-Free Kids Act of 2010 made many obesity prevention reforms to school environments, including improving the nutrition standards for school breakfasts and lunches. Given the large proportion of low-income students in our study population, changes in WIC food packages and school breakfast and lunch may have contributed to our findings (4). National and regional increases in breastfeeding rates have also been cited as potential contributors (4).

A major strength of this study was the large and representative sample (80% participation) of kindergarten students from a diverse metropolitan school district for 7 continuous years. Another strength was the linkage of BMI data with school district enrollment information, including FRPL status, disability status, and parent reported child race/ethnicity.

Several limitations to this study should be considered. Although previous research indicates that height and weight measurements collected by school nurses are reliable (29), lack of rigorous protocols, such as repeated height measures, introduces a potential for measurement error and could reduce BMI accuracy. Another limitation was the use of FRPL status as a proxy for SES, because this measure does not directly reflect parental income, education level, or family wealth (30). The true number of kindergarteners with disabilities was likely higher, because many children are not identified by the school district as having a disability until they are older. Because of this potential misclassification error, the associations between obesity and disability status are likely underestimated. In addition, including all disabilities in one category is not as useful as is an analysis that includes disability categories that are associated with obesity risk. Because this was a public school-based surveillance project, the data set excluded children who are exclusively home-schooled or who attend private schools, whose risk of obesity may differ with that of children who attend public school. This data set was also regionally specific and analyzed a population that had more Hispanic, American Indian, and low-income children than the rest of the nation, which may limit the generalizability of the results. However, the overall finding that obesity and severe obesity prevalence are no longer increasing in this age group is supported by other regional and national studies, lending confidence to our conclusions.
